# Investigation of Visceral Leishmaniasis among 192 Dog Carcasses Killed by Road Accidents in Khorasan Razavi, Northeastern Iran during 2014–2016

**Published:** 2018-11

**Authors:** Elham MOGHADDAS, Abdolmajid FATA, Mehdi ZAREAN, Majid DERAKHSHANI, Mahdi FAKHAR, Seyed Aliakbar SHAMSIAN

**Affiliations:** 1. Dept. of Parasitology and Mycology, School of Medicine, Mashhad University of Medical Sciences, Mashhad, Iran; 2. Cutaneous Leishmaniasis Research Center, Mashhad University of Medical Sciences, Mashhad, Iran; 3. Dept. of Microbiology, School of Medicine, Sabzevar University of Medical Sciences, Sabzevar, Iran; 4. Dept. of Parasitology and Mycology, School of Medicine, Mazandaran University of Medical Sciences, Sari, Iran

**Keywords:** *Leishmania infantum*, Dogs, kDNA, PCR, Iran

## Abstract

**Background:**

Visceral leishmaniasis (VL), so-called Kala-azar is a life threating parasitic infectious disease caused by *Leishmania* spp. *L. infantum* is the main causative agent for Mediterranean form of Kala-azar which is endemic in northeastern Iran. This study attempted to investigate existence of canine visceral leishmaniasis (CVL) in Khorasan Razavi.

**Methods:**

Between 2014 and 2016, tissue samples collected from spleen and liver of 192 stray dogs were examined to investigate existence of *L. infantum*. Kinetoplast DNA (k-DNA) PCR was performed to identify the species of parasites. The positive PCR products were sequenced in both directions to confirm the kDNA PCR results

**Results:**

Among samples obtained from 192 dogs, kinetoplast DNA of *L. infantum* was detected in two female dogs. *L. infantum* was confirmed by sequence analysis of PCR products.

**Conclusion:**

Our data confirm stray dogs play as potential reservoirs for VL in this province. Further investigation will be necessary to clear role of stray dogs in the transmission of *L. infantum* to human and domestic dogs.

## Introduction

Leishmaniasis is an infectious disease caused by protozoan parasites of the genus *Leishmania* (1, 2). It is transmitted by the bite of certain species of sand-flies as vector (3–5). Visceral leishmaniasis (VL) also known as kala-azar is a serious health problem in endemic area. The disease has huge importance for health care system throughout the world and it can be deadly without proper treatment (6). About 2 million new human cases are reported annually in 98 endemic areas in Europe, Africa, South America and Asia (1, 7). *L. infantum* is the main causative agent of VL in Mediterranean regions like Iran (8). There are several important endemic VL foci in Iran: Ardabil, Fars, Boushehr, Qom and northern Khorasan and some sporadic foci (9, 10). The results of a systematic review in Iran showed that the overall prevalence rate of canine visceral leishmaniasis (CVL) is 16% (9). During 1998–2006, approximately 2.056 cases of Human Visceral Leishmaniasis (HVL) were reported in Iran, 44.6% of them were reported from Ardabil. More than 90% of HVL cases are reported in children up to 10 yr old (11, 12).

Khorasan Razavi Province (Northeastern Iran) is an endemic focus for cutaneous leishmaniasis but recent studies showed sporadic cases of VL in this area These findings suggest the possible infection of VL reservoir in this area (13, 14). Dogs and red foxes are the main reservoirs host for *L. infantum*, but wolves and jackals can be suitable reservoirs too (15). Previous published study in this area was limited to symptomatic case and it was presented on clinical signs. No study was done on asymptomatic reservoirs in this region. Since in previous study *L. infantum* was isolated from dog with clinical presentations of VL, it was decided to continue this research (14).

## Materials and Methods

### Study area

**Fig. 1:**
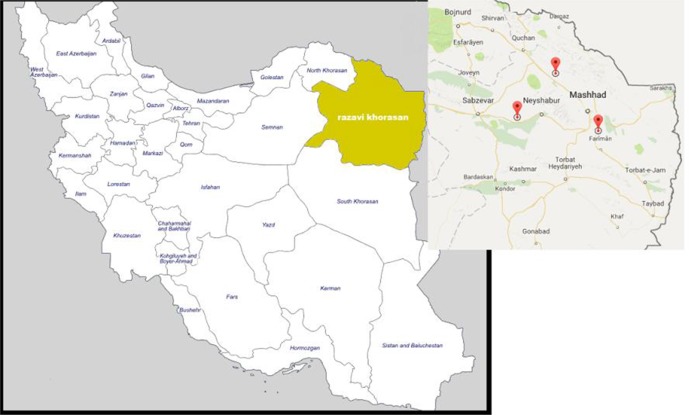
Three geographical regions in northeastern Iran collected carcasses of stray dogs, where the carcasses of stray dogs were collected

The investigation was carried out on dogs without clinical sign (asymptomatic) in Mashhad (capital of Khorasan Razavi Province) which is the second most populous city in Iran (Fig. 1). This province is located at 36.20° North latitude and 59.35° East longitude and stands on the northeast of Iran with 71.9% are living in the urban areas and 28.1% in rural areas.

### Sampling

This cross-sectional study was performed from Jun 2014 to Apr 2016. Overall, 192 stray dog carcasses killed due to road accident, were collected. All sampling was done by a veterinarian and postmortem changes were seen carefully. Dead time was estimated between 12 and 24 h ago. These roads were located in north, south and west of Mashhad City (Fig. 1). A questionnaire was completed for each dog, recording clinical signs of VL such as skin lesions, cachexia, and hepatosplenomegaly. Spleen and liver samples were obtained and kept in bottle containing 70% ethanol. They were transported to the molecular laboratory at School of Medicine in Mashhad University of Medical Sciences.

### Molecular identification

#### DNA extraction

Despite we have liver and spleen samples, DNA extraction was done on spleen only. Because digestion of liver was very difficult by proteinase K and it needs so much of this enzyme. DNA was extracted from all spleen samples based on method (16). Spleen samples were homogenized with 200 μl lysis buffer [50 mM Tris–HCl (pH = 7.6), 1 mM EDTA and 1% Tween 20%] and 10 μl of proteinase K solution (containing 20 mg of the enzyme/ml), then incubated at 37 °C overnight and after that 200 μl of a phenol, chloroform, iso-amyl alcohol mixture was added. After strong vigorous shaking the mix, the tube which was holding the mix was centrifuged (10000 gr for 10 min) and then the DNA in the supernatant solution was precipitated with 400 μl cold, pure ethanol re-suspended in 50 μl double distilled water and then stored at 4 °C until it could be tested. It was re-suspended in 100 μl sterile distilled water and stored at 4 °C (16). Positive control that contained the DNA from the reference strain was prepared from Regional Leishmaniasis Diagnostic Reference Lab (RLDRL) in Department of Parasitology and Mycology, School of Medicine, Mazandaran University of Medical Sciences.

#### PCR Amplification

The Kinetoplast DNA (k DNA) of *Leishmania* was amplified by RV 1 (5-CTT TTC TGG TCC CGC GGG TAG G-3) and RV 2 (5-CCA CCT GCG CTA TTT TAC ACC A-3) primers that amplify a 145-bp sequence from the *Leishmania* kDNA mini-circles. The PCR products were segregated in 2% agarose gel and stained with ethidium bromide, visualized under ultra-violet trans-illumination, and sized by comparison with a 100 bp ladder. Each sample found PCR-positive for *Leishmania* DNA was then evaluated using the PCR species-specific primers LINR4 and LIN17 to identify the species of *Lieshmania* parasite (17).

##### DNA Sequencing

PCR amplification of the kDNA minicircle gene from 2 samples was subjected to sequencing by MWG (Germany) by the primers employed. The GenBank database was searched for similar sequences using BLAST (National Center for Bio-technology Information; https://blast.ncbi.nlm.nih.gov/Blast.cgi) and the output was analyzed to find a significant homology.

#### Ethical approval

This study was reviewed and approved by the Ethics Committees of Mashhad University of Medical Sciences, Iran. (N= IR.MUMS.fm.REC.1395.298)

## Results

**Fig. 2:**
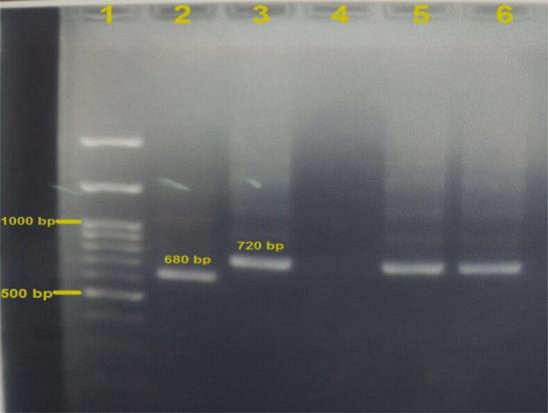
Gel electrophoresis of PCR products of *Leishmania* kDNA using LINR4 and LIN17 primers from spleen of the stray dogs in Khorasan Razavi, Iran

This study carried out on 192 dogs (137 males and 55 females), under 3 yr old. PCR results confirmed CVL infection by *L. infantum* in 2 dogs in Neyshabur road. They were both females. In neither of the 2 infected dogs any sign of CVL was observed. Expectation of pattern bands of *Leishmania* spp. were for *L. infantum* at 720 bp, *L. major* at 680bp and *L. tropica* at 780 bp (18). Amplification reactions in 2% agarose gel electrophoresis using a 100bp DNA ladder are shown in Fig. 2. To confirm and complete the identification of samples, selected two PCR products were sequenced and submitted in the GenBank database (https://www.ncbi.nlm.nih.gov/nuccore/) with accession numbers: KM350534.

**Lane 1:** DNA size marker 100bp, **lane 2:** *L. major* (positive control standard = 680bp), **lane 3:** *L. infantum* (positive control = 720 bp), **lane 4:** Negative control, **lanes, 5, 6:** *L. infantum* isolates obtained from spleen of the stray dogs

## Discussion

Mashhad is an attractive city for religious people and welcomes approximately 25 million tourists every year. This subject indicates high demand of attention from healthcare system as a strategic area. In general, control of zoonotic visceral leishmaniasis, important control programs are based on human case detection, treatment and elimination of animal reservoirs (19).

Determining the prevalence of canine leishmaniasis as source of visceral leishmaniasis is one of the necessities in control and prevention of disease (20).

Infected dogs even though asymptomatic, are the putative sylvatic animal reservoirs. It was determined the potential role of asymptomatic infected dogs as reservoirs to be very importance (21). Therefore examination of asymptotic dogs is important to identify *L. infantum* infection (18). It is interesting to note that in our study both cases of infected dogs had any clinical sign of CVL. Therefore diagnosis of VL is not confirmed by observing of clinical presentation. Epidemiological survey in every region should be to combine by serology or molecular approaches. PCR was more accurate in identifying canine visceral leishmaniasis (CVL) compared with serologic methods (14, 22, 23). Sometimes infected dog maybe had specific antibodies against *L. infantum* with negative microscopically results (14).

North Khorasan was an important endemic region for Kala-azar (24, 25). In a study on 104 patients from Khorasan Province, more than 55% of them were from North Khorasan and about 21% of them from central Khorasan including Mashhad suburb (26). Recent reports also indicating the Mashhad district is still a focus for human VL (27). In present study, we found *L. infantum* in 2 dogs which aligned with other studies (28). This result implies probability of spreading risk of VL in this area; maybe we have the danger of human cases in future.

In one study, direct agglutination test (DAT) was used to determine the seroprevalence of visceral leishmaniasis in parts of Iran (24), DAT is simple and highly sensitive (92%–100% technique) (29). Therefore it is commonly used in epidemiologic studies and diagnosis of leishmaniasis, but statistical analysis showed the more specific detection of *L. infantum* when using real-time PCR assay (25). There have been cases of cutaneous leishmaniasis (*L. tropica*) spread via blood to visceral organs (12, 30), in these cases the serologic tests would appear positive but it does not show *Leishmania* species.

Investigation of VL on dog carcasses which killed on the roads of Khorasan Province had some limitations in this study. The limited number of population study, difficulty on exact examination of signs in carcasses killed 2–3 d before observation in different climate conditions and difficulty in diagnosis were the main problems. Postmortem change is an important phenomenon considered in future studies. We suggest using live dogs instead of dead cases (25). Liver dog samples digested in proteinase K solution hardly and we had to use spleen samples for DNA extraction. In one study on infected dogs, it was shown detection of kDNA by PCR from skin samples is better results than tissue samples in symptomatic animals (31). It is noteworthy almost infected dogs are asymptomatic (8, 9, 10, 32).

In another study on feline visceral leishmaniasis it was found the possible role of cats as VL reservoirs for humans (33, 34). Considering low prevalence of infection among dogs, it was suggested the possible role of feline and canine as reservoirs for infection (35). Several studies report infected cats do not have the potential role in *L. infantum* transmission to human (25, 37), however, some articles in conflict with this opinion (37, 38).

Therefore more research needs to be undertaken to find such an association between feline leishmaniasis and human infection. In accordance with other studies domestic dogs, due to adjacency to human, may be remarkable reservoirs (24, 25). It can be suggested to clear role of domestic dogs in Khorasan province in human infection in future studies.

## Conclusion

The existence of *L. infantum* in stray dogs in northeast of Iran implies probability of danger of visceral leishmaniasis to human. Our data confirm stray dogs at least play as potential reservoirs for VL in this province, consequently control measurement are required. This research will serve as a base for future studies on dogs and other possible sources of infection and highlights the importance of monitoring the surveillance system by health authorities in this region. Moreover, further investigation will be necessary to explore the possible reservoir and vector hosts in the area.

## Ethical considerations

Ethical issues (Including plagiarism, informed consent, misconduct, data fabrication and/or falsification, double publication and/or submission, redundancy, etc.) have been completely observed by the authors.
